# Sensing Systems in Construction and the Built Environment: Review, Prospective, and Challenges

**DOI:** 10.3390/s23249632

**Published:** 2023-12-05

**Authors:** Amin Malekmohammadi, Nima Farzadnia, Amir Hajrasouliha, Ashley Lyn Mayer

**Affiliations:** 1 Department of Computer and Electrical Engineering, California State University Bakersfield, Bakersfield, CA 93311, USA; 2 College of Engineering and Mines, University of Alaska Fairbank, Fairbanks, AK 99775, USA; 3 Department of City & Regional Planning, California Polytechnic State University, San Luis Obispo, CA 93407, USA; asmayer@calpoly.edu; 4 Department of Computer Engineering, California Polytechnic State University, San Luis Obispo, CA 93407, USA

**Keywords:** sensing systems, smart sensor, construction

## Abstract

This article is a comprehensive review of state-of-the-art sensors of the built environment, applicable in construction, structural engineering, management, and planning industries. This review is framed within the technical definition of sensing systems and their components. Existing sensors are listed and described in two broad categories of structural health monitoring (SHM) and building environment monitoring (BEM). The SHM systems are used for monitoring the long-term performance of structures, such as bridges and buildings. BEM systems are employed to ensure the safety and comfort of the built environment’s occupants, as well as the general monitoring of the environment for any required maintenance. The applications and implementation challenges of both systems are discussed, with emphasis on common sensing system limitations such as energy supply, packaging, network layout, and performance validation. Finally, the prospect of sensing systems as part of a digital twin that incorporates multifunctional sophisticated monitoring systems and intelligent analysis methods is discussed.

## 1. Introduction

### 1.1. Definition of Sensing Systems

Sensing systems are subcategories of non-destructive techniques (NDT) used for the continuous supervision and maintenance of civil structures. In general, NDTs have a wide variety of implementations, where some can be in a controlled testing environment or in situ (on-site) assessments of the properties of a structure without affecting the functional value. In this paper, two main applications, namely, structural health monitoring (SHM) systems and building environment monitoring (BEM) systems, will be reviewed and discussed. The SHM systems are best suited for monitoring the long-term performance of structures, such as bridges and buildings, where structural health is crucial to the functional value. The SHM implements a sensing network that takes measurements of a structure’s physical and mechanical properties and a data analysis system to identify any damage or irregularities within the structure [1]. The SHM systems allow for the early detection of damage, degradation, and corrosion, even in areas not visible to the human eye. However, these methods do not substitute for regularly scheduled maintenance plans or compensate for poor architectural or mechanical designs. The critical components of a sensing system are the data-acquisition equipment (DAE) and the data-acquisition system (DAS). The DAE comprises the hardware and sensors needed to measure, store, and communicate the acquired data. The on-site sensors convert physical measurements into electrical signals to transmit to the off-site DAS for processing. In the DAS, the data is analyzed by an algorithm to extract the relevant features and identify significant results. Monitoring previous and real-time data can also allow the algorithm to predict future advancements in the current structural conditions. With these predictions, necessary maintenance can be performed to ensure the continuing functionality of a structure.

The SHM systems provide a way to monitor, in great depth, the current conditions and predict future conditions of a structure, which, with preemptive actions, can increase the design life, reduce rehabilitation costs, and provide public safety [2]. Recent advancements in SHM systems include integrating a sensing network into the construction of civil infrastructure and the various connection networks of these implementations. Such sensing systems have recently attracted enormous attention with the trend for additive manufacturing. That includes the in situ assessment of material properties before, during, and post-printing for optimized printability and the printed structure’s performance [3]. Such sensors are used to measure the properties of the printing materials and the printing parameters in real-time through intelligent control systems. The control systems facilitate the collision-free performance of robots as well as in situ modification of the printing material. On the other hand, BEM systems are employed to ensure the safety and comfort of building occupants, as well as the general monitoring of the environment for any required maintenance. This is fulfilled using a wide variety of different sensors that will be discussed.

### 1.2. Construction: Process, Materials, and Defects

The construction of a civil structure can use a wide variety of materials, but the most commonly implemented are the most cost-efficient and functional. Wood, steel, concrete, and asphalt are some of the many common materials that make up a large portion of our civil structures today. These materials, however, are subject to degradation over time and usage due to various predictable and unpredictable environmental factors. The well-being of these materials can determine if a structure is functional and safe for use, so it is important to ensure the internal strength of the elements.

Wood, common in construction applications for buildings, bridges, and many more, is highly susceptible to environmental and mechanical factors and internal defects that may influence its structural performance [4]. The inner strength of wood is dictated by the direction of stress that is applied, the internal and external humidity, and the length of time in which a load is applied [1]. Wood, in particular, is also vulnerable to many environmental factors, such as rot and insect infestation, which may remain undetected until the damage is too extensive for repair. These factors, especially over time, can lead to structurally fatal defects within the wooden components that may call for the comprehensive rehabilitation of a structure. Asphalt and concrete are other widely used materials for infrastructures such as roads and bridges, and the health of such infrastructure is crucial to its functional value. These structures endure large amounts of stress and heavy loads and are prone to damage over time [5]. Detecting any defects in the structure could prevent damage such as cracks and corrosion in bridges as well as potholes and sinkholes in roads by scheduling maintenance at an early stage. For example, with surface infrastructures being a major form of transportation, there is much dependence on the functionality of these structures. Small-scale rehabilitation for minor damage could potentially prevent an entire shutdown of a fatally damaged roadway [6].

Monitoring the health of these structures that are guaranteed to have defects over time is an essential mechanism for maintaining a functional civil design. Sending out humans for maintenance requires a lot of time and labor. It is not always practical, as some internal measurements require invasive techniques that may hinder the usage of the structure for a limited amount of time. Implementing sensors within these structures eradicates the need for a large amount of labor to take these measurements and allows for continuous monitoring of structural health and the environment that directly affects the service life of the structure [7]. Sensing systems can monitor material attributes that may cause damage that would require heavily invasive measurements with a manual labor team.

### 1.3. Sensing System Components

Commonly, the sensing equipment implemented in sensing systems is used to measure structures’ physical and performance characteristics. These sensors can detect physical properties and translate them into mechanical properties to be measured. Many of these sensors incorporate at least one standard hardware that can take measurements or generate signals and, create a cooperative sensing network. The common measurement instruments found in a basic sensing system can be of the following categories [1]:

- Transducer: Transducers are pieces of hardware that are capable of converting one form of energy into another. In the SHM systems, these can be categorized as a sensor or an actuator based on the input or output of an electric signal.
- Sensor: A sensor is a type of transducer that reacts to a stimulus by converting the stimulus into an electrical signal. This electrical signal is then processed and transmitted to the database, or DAS.
- Section 2 and Section 3 of this review describe different types of sensors.
- Actuator: An actuator is a type of transducer that converts an electrical signal into a different form of energy to be applied to a structure or structural element. These predefined excitations include but are not limited to, mechanical strain, vibrations, and ultrasonic signals. Actuators require an electrical energy source and a control signal to operate.
- Gauge: An instrument that measures the amount of a property, usually through a visual display on mechanical instruments. These instruments provide quantifiable data measurements of the property under review. Examples are pressure gauges, vibrating strain gauges, and dynamic strain gauges.

Recent advancements in sensing systems have integrated a wireless sensor network (WSN) or wireless smart sensor network (WSSN) that allows the transmission of sensor data through a wireless network. Compared to the wired communication networks of a typical sensing system, these ‘smart’ sensing systems require a few more hardware elements to increase the scalability of the network. These systems use the typical hardware involved in a sensing system, along with the following additional hardware [7]:

- Global positioning system (GPS): A position tracking system to identify the locations of the placed sensors and where the data they transmit originates from. These instruments allow for localization of damage detection and locating where maintenance may be required.
- Analog to digital converter (ADC): Converts the analog electrical signal from the transducer into a digital signal that can be transmitted across networks. These digital signals are data bits, which can be communicated and translated between technological systems.
- Radio transceiver: A network transceiver that connects an internal sensor antenna to an external one that connects the sensor node to the greater network, such as the DAS. Transceivers may also support communication between sensors within the same network for cooperative sensing.

All sensing systems require a connection to a power source and some means of transferring the data from the sensors to the database, whether through a wireless network or ethernet cables. The various sensing components can be incorporated to formulate a complex sensing network, which may be unique to the structure that is being monitored. Depending on what properties are under review and what resources are available, the sensing network may or may not implement all these components. Either way, the network undergoes rigorous design to find the most effective sensing method and respective units for its application. Together, these hardware components create the physical network needed to monitor the properties of the intended structure.

### 1.4. Modeling Processes

The modeling process for a sensing system typically calls for a 3D model of the structure to be monitored. Using a 3D image-based model allows for the location of any damage detected to be recognized with a visualized specification rather than a verbally described place. The model creates a virtual replica of the structure and the sensing network implemented throughout it. This way, the sensor locations can be planned (before implementation) and identified (after implementation) with a visual description of the components they monitor. The data reported from the implemented sensors is transmitted to a database, analyzed for relevant details, and the model is updated according to the reported measurements. These updates can happen in real-time and allow for continuous monitoring of a physical structure through a virtual environment. The data can be localized to a specific region, and the model can help identify where any maintenance or rehabilitation is needed. The model of the structure, paired with some algorithm or AI for data analysis, creates a Digital Twin of the system that simplifies remote monitoring of structural health and the building environment.

## 2. Sensors for Structural Health Monitoring

### 2.1. Fiber Optic Sensors

**Figure 1:**
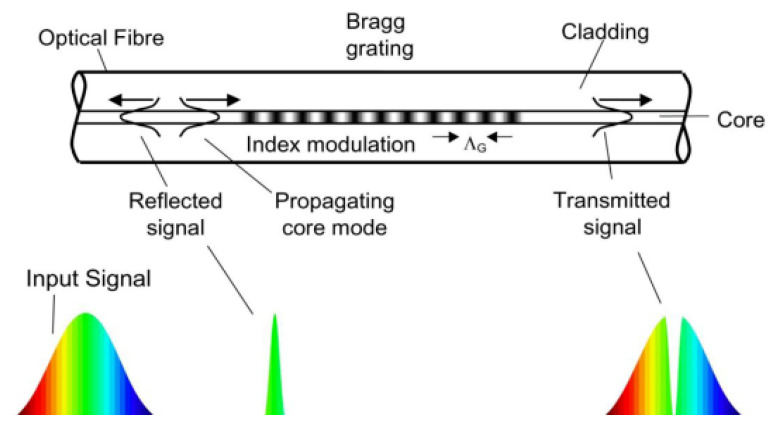
Schematic diagram of an FBG [8].

Fiber optic sensing technology is an optic measurement strategy that utilizes light waves and fiber-optic channels to measure the physical properties of a structure. The characteristics of light, such as its wavelength and frequency, are altered by the physical characteristics of the measured structure. Compared to a control waveform of a healthy structure, changes in the light properties can indicate a change in the structure’s internal physical properties. Palma and Steiger (2020) [1] describe the instrumentation for this system as optic fibers that can operate as a “both sensor and signal transmission medium”, where one optic fiber can house up to 10 sensors with a process known as multiplexing. The sensors embedded in the optic fibers reflect only a specific wavelength and allow others to transmit, known as fiber Bragg’s gratings (FBG), Figure 1 [8], With multiple sensors in a single fiber and the dual-function of the optic cables, this technology allows for long-range data transmission and high-frequency measurements. Banda et al. [9] employed analytical modeling, simulation, and experimental test of a structural damage sensing system based on fiber Bragg grating strain sensors to predict structural decay. The authors reported that the strain measured from the FBG sensors could be used to predict the damage of locations even if those locations are not instrumented if the sensor placement is optimized. Such fibers are best suited for measuring strain and temperature because the light waves are hardly affected by external variables such as electromagnetic or vibrational interferences. However, the FBG sensors are affected by temperature, but this can be accommodated through additional measurements and data adjustment. The optic fibers are delicate and complex, requiring careful, sophisticated installation and implementation. Despite the high production costs, the fiber optic method has been popularized as a durable and efficient measurement system for long-term structures [10,11].

Another type of fiber optic sensor that plays a crucial role in structural health monitoring is the fiber optic demodulator. Demodulators are essential components in optical fiber sensor systems, facilitating the extraction of information encoded in the optical signals transmitted through the fiber. These devices are designed to recover the modulation imposed on the light wave, allowing for the measurement of various physical parameters.

In the context of monitoring structural health, fiber optic sensors are often used to measure both temperature and strain. However, to decouple the effects of temperature and strain induced by external loads, innovative techniques are employed. One common method involves using a combination of different fiber optic sensor types, each sensitive to either temperature or strain. For example, a fiber Bragg grating (FBG) sensor is highly sensitive to strain variations, while an interferometric sensor, such as a Fabry–Perot interferometer or a Mach–Zehnder interferometer, can be specifically designed to be more responsive to temperature changes. By strategically placing and integrating these sensors within the structure, it becomes possible to separate the contributions of temperature and strain, enabling more accurate and reliable monitoring of structural conditions under varying environmental and loading conditions. This approach allows for the extraction of precise and independent measurements of temperature and strain, enhancing the overall effectiveness of fiber optic sensor systems in structural health monitoring applications [12,13].

### 2.2. Piezoelectric Sensors

Piezoelectric sensors play a crucial role in structural health monitoring (SHM), providing a versatile and effective means of assessing the integrity of various civil and mechanical structures. These sensors operate on the principle of piezoelectricity, where certain materials generate an electric charge in response to mechanical stress. In the context of SHM, piezoelectric sensors are often employed to monitor structural vibrations, strain, and impact events. One of the key advantages of piezoelectric sensors is their high sensitivity, allowing for the detection of subtle changes in a structure’s behavior. These sensors can be strategically placed on or within a structure, such as bridges or buildings, to capture dynamic responses and identify potential issues, including fatigue, cracks, or other structural anomalies. The real-time monitoring capabilities of piezoelectric sensors make them valuable tools for ensuring the safety and reliability of critical infrastructure.

Piezoelectric sensors for structural monitoring come in various types, each designed to address specific monitoring needs. One common type is the accelerometer, which measures accelerations and vibrations in structures. Accelerometers based on piezoelectric materials are ideal for capturing dynamic responses, enabling the detection of vibrations caused by environmental factors, traffic loads, or other external forces. Another type is the piezoelectric strain sensor, which measures changes in strain or deformation within a structure. These sensors are often used to identify subtle shifts in structural elements, helping to pinpoint areas experiencing excessive loads or stress. Additionally, piezoelectric pressure sensors can be employed to monitor changes in pressure within structural components, providing insights into load distribution and potential structural weaknesses. The versatility of piezoelectric sensors allows them to be customized for specific applications, and advancements in sensor technology continue to lead to the development of new sensor types that further enhance their capabilities in structural health monitoring. The ability to choose from various sensor types allows engineers and researchers to tailor monitoring systems to the unique requirements of different structures and environments, contributing to more effective and targeted structural health assessments.

Furthermore, piezoelectric sensors offer benefits beyond their sensitivity and real-time monitoring capabilities. They are relatively compact and lightweight, making them easy to integrate into existing structures without causing significant alterations. This characteristic is particularly advantageous for retrofitting older infrastructure with monitoring systems. Additionally, piezoelectric sensors can be part of a broader sensor network, working in conjunction with other types of sensors to provide a comprehensive understanding of a structure’s condition. The data collected from these sensors can be analyzed to assess structural health, predict potential issues, and guide maintenance activities, ultimately contributing to the longevity and safety of civil and mechanical infrastructure [14,15,16,17,18,19,20,21].

### 2.3. Piezoresistive Sensors

Piezoresistive sensors operate similarly to piezoelectric sensors. These sensors, however, are purely vibration-based and are best suited for flexible structures that require low-frequency vibration measurements [22,23]. In the vibration-based approach, the identification of the “existence, location, type, and extent” of structural damage depends on the relationship between the measured and “model parameters” [1]. This type of analysis requires measurements to be taken on the frequency response of a healthy structure to compare data to and differentiate between healthy and damaged structural members. In the low-frequency responses of a structure, actuators, such as shakers, are used to apply a forced vibration or excitation to the structure. These vibrations impact all modes of the structure equally, allowing for a global damage detection technique. These shakers can operate in a constant frequency white noise mode or sine sweep mode, where the actuator alternates between low and high-frequency excitations. The response of the vibration is captured by the sensors and transmitted for comparative analysis. The system can also use ambient excitations, where the environment applies the vibrational force. Still, this method requires complex modal parameters, the ability to operate without knowing when the system is to be excited, and the identification of low amplitude vibrations. This can make it difficult to extract significant and relevant data from the responses. Kuo et al. [22] studied the problems associated with temperature stability and time drift when using piezoresistive sensors in bridges. Their results showed the sensors with higher boron doping concentrations and lower stiffness were less sensitive to temperature variation and time drift problems, respectively. Overall, piezoresistive sensors in a low-frequency application allow for possible damage detection in a structure through global changes.

**Figure 2:**
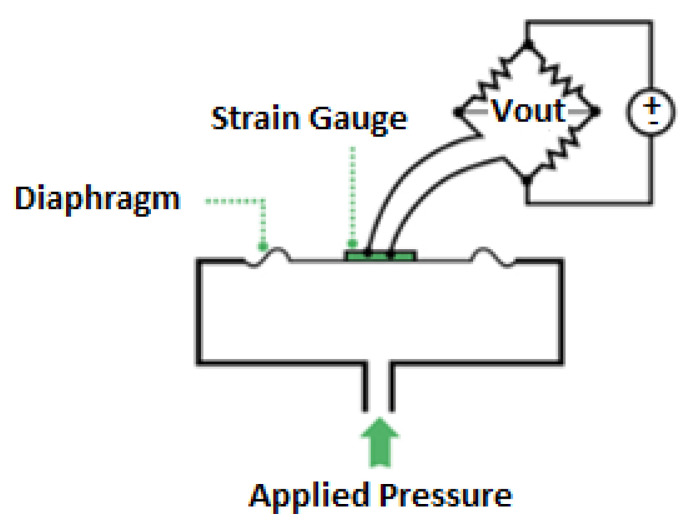
Piezoresistive strain gauge measurements are made using a Wheatstone bridge circuit [24].

Piezoresistive sensors exhibit both advantages and drawbacks that impact their suitability for various applications. One of the significant advantages is their sensitivity to changes in strain, making them effective in measuring deformation and stress in structural elements. Their high sensitivity allows for precise detection of subtle variations in the material, providing valuable insights into structural health. However, piezoresistive sensors are susceptible to environmental factors, with moisture being a notable concern. Moisture can affect the resistivity of the sensing material, leading to inaccuracies and reducing the sensor’s reliability over time. Consistency is another consideration, as piezoresistive sensors may exhibit variations in performance due to manufacturing tolerances. Achieving consistent results across sensors can be challenging, impacting the overall reliability of a monitoring system. On the positive side, these sensors often offer good linearity in their response to applied strain, simplifying the interpretation of data. Additionally, piezoresistive sensors can provide accurate measurements when properly calibrated, offering valuable data for structural health monitoring applications when environmental conditions are carefully controlled. Despite these challenges, ongoing research and technological advancements aim to mitigate drawbacks and enhance the overall performance of piezoresistive sensors in structural monitoring contexts. Figure 2 shows a typical piezoresistive strain gauge using a Wheatstone bridge.

### 2.4. Nanosensors

Nanosensors refer to sensors that exist and can take measurements on the nanometer scale. These sensors are mainly piezoelectric materials that typically take the form of fine powders or liquids, as well as nanofibers that can be applied to a structure to measure its material performances or conditions [25,26]. Nanosensors have been integrated into a large variety of forms, including smart paints. The smart paint sensor is made from a mixture of piezoelectric material and polymers and is applied to a surface like paint. The paint can also be made with a mixture of epoxy resin and carbon nanofilaments that increase the sensor’s sensitivity to vibrations and its conductivity. The sensor is flexible and even suited for curved surfaces, welded joints, or complex geometries [7]. Operating in a similar fashion to a traditional sensor, the smart paint can detect damage, impacts, and other dynamic responses through voltage signals generated between a silver paste and the surface to which the paint adheres. These sensors are easy to implement, low cost, and require minimal power to operate.

Carbon nanofilaments such as carbon nanofibers, nanotubes, as well as graphene pellets are other commonly used nanosensors in construction [27]. Using such materials can replace the disadvantages of traditional sensors like strain gauges. A small percentage of such materials (1% of the total volume) can increase the ductility of concrete by 100 times [28]. This will provide a continuous path of conductivity that can transfer the change in the electric resistance when an external load is applied to sensors. The deformation in members can be measured by correlating the electric resistance and stress.

### 2.5. Laser Displacement Sensors

Laser displacement technology is a method of optic-based measurements that utilizes a laser beam to measure the distance and displacement of a structure over time. The laser displacement system consists of mounted equipment that takes periodic scans of a structure and measures the distance between the components. The distance is calculated using either a “time-of-flight measurement of a laser pulse or phase comparison between as transmitted and reflected continuous laser beam” [1]. The distance measurement is compared to previously stored data to detect any structural position differences. This method is generally not suited to detect any minor damage or deformations that would be smaller than a centimeter, but it is able to assess larger, more significant deteriorations of a structure. One major advantage of this method is the ability to operate independently of a natural light source and the direct access to the measured structure, despite equipment costs and lower accuracy relative to alternative methods. This method can also be applied to the displacement measurement of large-scale structures [29]. Park et al. [30] proposed a wireless displacement measurement system for a large-scale irregular structure to monitor the vertical displacements of the truss elements during construction.

### 2.6. Rotation Measurement Sensor

Rotation measurement sensors play a critical role in various applications where monitoring and quantifying angular displacement or rotational motion is essential. These sensors are designed to detect and measure the extent of rotation around a specific axis. One common type of rotation measurement sensor is the gyroscope, which utilizes the principles of angular momentum to determine the rate of rotation. Another example is the rotary encoder, which converts mechanical rotation into electrical signals. These sensors find widespread use in industries such as aerospace, automotive, robotics, and manufacturing, where precise control and feedback on rotational movements are crucial. The data obtained from rotation measurement sensors can be utilized for tasks such as navigation, stabilization, and positional feedback in machinery and equipment. The continuous advancements in sensor technologies, including the integration of micro-electromechanical systems (MEMS) and fiber optics, contribute to the development of more accurate and compact rotation measurement sensors, expanding their applications in diverse fields [31,32].

## 3. Sensors for Building Environment Monitoring

The many aspects of a building’s internal environment can be measured using a wide variety of different sensors. These sensors can be used to ensure the safety and comfort of the building occupants, as well as general monitoring of the environment for any required maintenance [33]. Below, widely used sensors to measure environmental variables and their working mechanism are listed.

### 3.1. Temperature Sensors

**Figure 3:**
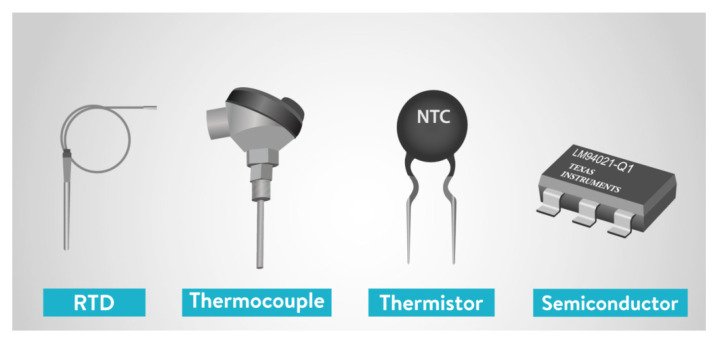
Types of temperature sensors [35].

Temperature sensors measure the ambient temperature of the environment they are placed in through different hardware elements. Available temperature sensors meet the requirement of temperature monitoring in buildings; however, their performance in terms of simplicity of operation, accuracy, range, and response time may vary [33,34]. Figure 3 shows different types of temperature sensors.

Common types of temperature sensors are:

- Semiconductor-based sensors: Constructed with identical diodes that use temperature-sensitive voltage compared with current conditions to detect changes in atmospheric temperature.
- Thermocouple: Two wires of different metals are placed at different points, where a change in atmospheric temperature will reflect as a change in voltage between the two wires.
- Resistance temperature detector: Constructed as a film or wire wrapped around a ceramic or glass core, and a change in the element’s electrical resistance reflects a temperature change. These tend to be the most accurate but also can be the most expensive type of sensor.
- Negative temperature coefficient thermistor: These sensors provide a high resistance in low environmental temperatures, and the resistance drops quickly as temperature increases. Reflects temperature changes quickly and accurately.

### 3.2. Humidity Sensors

**Figure 4:**
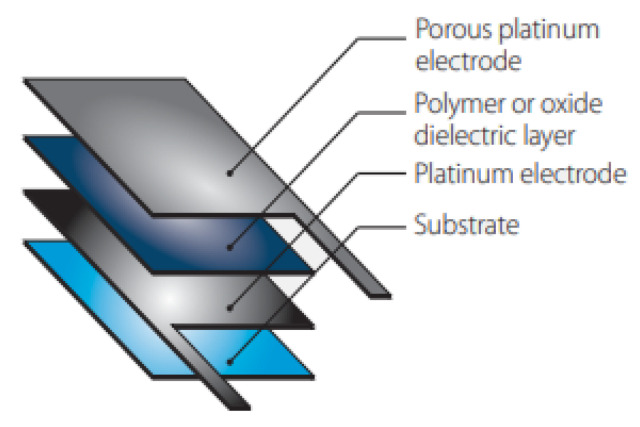
Capacitive humidity sensor [37].

**Figure 5:**
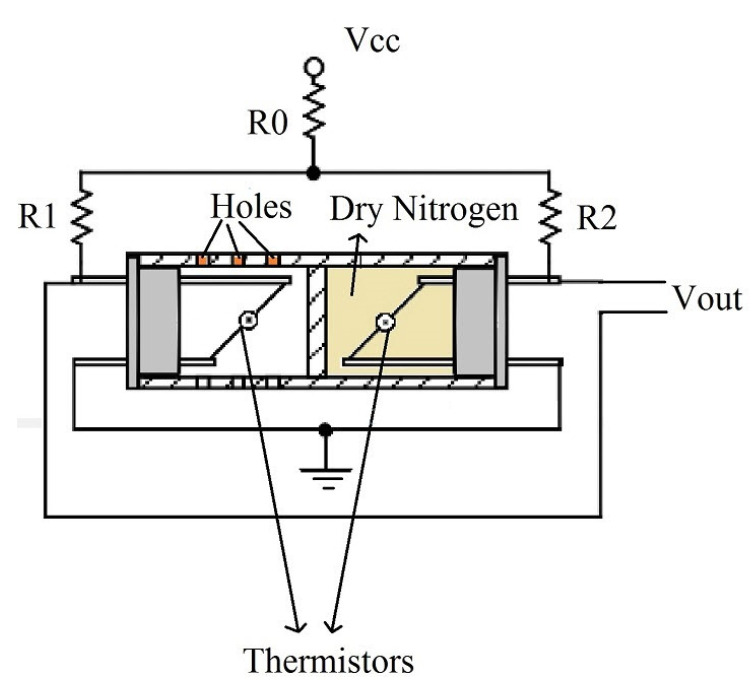
Thermal humidity sensor [37].

Humidity sensors are used to measure the amount or percentage of water vapor in ambient air. They can also be used within a structure to continuously measure the internal humidity, evaporation rate, and water penetration levels [36]. Figure 4 shows a capacitive humidity sensor. Three common types of humidity sensors are:

- Capacitive: The sensor uses a capacitor and water vapor to measure humidity levels. The capacitor has a porous dielectric core surrounded by two electrodes. The voltage across the capacitor changes when water vapor is present at the electrodes.
- Resistive: A similar operation to the capacitive sensor, this sensor uses an electrical charge to measure the humidity levels of the external environment. They use ion salts to measure the voltage difference across the electrodes and are generally less accurate than the capacitive sensors.
- Thermal: As illustrated in Figure 5, two matched thermal sensors, one coated in dry nitrogen and the other in ambient air, conduct electricity based on the humidity in the environment. The difference in electricity between them calculates the humidity reading of the environment.

Some of these sensors require complex circuit design as well as regular calibration. Those are mainly categorized under expensive sensors such as capacitive sensors. On the other hand, other sensors, such as resistive sensors, have a low cost but offer a narrow measurement range and are less accurate but have a good response time.

### 3.3. Motion/Occupancy Sensors

Motion and occupancy sensors use thermal detection to record the placement and movement of people within a space. These sensors operate by emitting ultrasonic waves or radio waves and detecting the time that it takes for the waves to bounce back to the emission spot. When a person or persons enter the sensor’s field of view, a portion of the emitted waves bounce back to the sensor before the rest, indicating there is an object in view. The proper use of such sensors in buildings can lead to energy savings, improved climate control, and higher building security [38,39]. At the same time, no images or personal information is stored or transmitted, making these sensors privacy-compliant. Common types of these sensors are:

- Motion sensors/passive infrared: These sensors use heat maps of peoples’ body heat in the sensor’s field of view. These sensors monitor continuously and can report the occupancy and movement of people. They can have a 180-degree or 360-degree field of view, depending on their location of implementation (underside of a table or desk versus on the ceiling). They are unobtrusive, cost-effective, easy to install, and low maintenance; however, they are only suitable for short-distance measurements (up to 10 m) [40].
- Time-of-flight sensors: These sensors emit an infrared light beam that reflects off an obstruction, such as a person, and back to the sensor. The time it takes for the light to return to the sensor reflects the distance and movement of the object reflected off. These sensors can determine if someone is moving towards or away from the sensor, allowing the detection of entry and exit, as well as the flow of foot traffic in an area.
- Infrared array sensors: These sensors use temperature measurements to detect objects (moving or motionless), temperature distribution, and moving direction. The objects must be closer to the sensor for more accurate temperature readings.

### 3.4. Contact Sensors

Contact sensors in buildings operate as a two-piece system that detects the magnetic field between the pieces to determine whether they are in contact. This sensor is usually used as a mechanism to determine if a door or window has been opened. On a door, one piece of the sensor is placed in the door frame, and the other is placed on the door itself. When the door is open, the magnetic field is disturbed, and the sensor transmits a corresponding signal. These sensors can be very useful in maintaining safety and observing the activity within a building.

### 3.5. Gas/Air-Quality Sensors

Air quality sensors monitor changes to the air quality and detect the presence of various gasses (hazardous and safe) in an environment. This device is useful for creating and maintaining a safe and healthy environment in buildings. The three most common air quality sensors are:

- Oxygen: An electrochemical sensor that can detect any gasses that can be reduced electrochemically or oxidized. Maintaining healthy oxygen levels is vital to the health and well-being of the occupants.
- Carbon monoxide: An electrochemical sensor that works similarly to the oxygen sensor. Carbon monoxide is a hazardous, invisible gas that is a large safety concern if left unattended in an occupied building. It is important to monitor the levels of this gas to protect the building community [41].
- Carbon dioxide: An infrared detection sensor that transmits an infrared light beam through a light tube, then detects how much of the beam’s energy levels remain (or are lost). This energy is then calculated into how much carbon dioxide is present in the air [42]. In highly insulated spaces, high carbon dioxide levels cause stale and stuffy environments, where occupants have complained of fatigue and headaches, affecting comfort and productivity.
- Smoke sensors: These sensors detect levels of airborne particulates and gasses, and recent developments allow for the notification of any issues immediately.

### 3.6. Electrical Current Monitoring Sensors

Electrical current (CT) sensors measure the energy consumption of a circuit in real time. This allows for identifying areas where energy is being wasted or there are abnormal operating conditions. To prevent energy waste, assets can be powered off when they are not necessary. Identifying any abnormal operations of a circuit can be a sign that maintenance is needed in that specific area. The different types of CT sensors are:

- Split core: These sensors can be opened and fitted around a preconfigured conductor in a circuit. These are ideal for existing circuits.
- Hall effect/DC: These make use of the Hall effect to measure AC and DC current by measuring the changing voltage of a device in a magnetic field. The Hall effect is the electromagnetic phenomenon that occurs when an electric current flows through a magnetic field, generating a voltage difference across a capacitor [43]. An open loop type of this sensor is compact, low-cost, and accurate. A closed-loop type of this sensor offers quick response and consistency of results across environmental temperatures (low-temperature drift).
- Rogowski coils: These are a type of flexible current transformers that consist of a thin coil that is wrapped around a conductor. These are easy to install on pre-existing circuit configurations and are snapped closed [44].
- Solid core: Best for new installations, these sensors are complete loops with no way of opening and are accredited for their high levels of accuracy.

## 4. Data Processing and Analysis of Data

The fundamental construction of a sensing system consists of the sensor configuration and the data processing/analysis methods. The sensor configuration is primarily composed of the hardware-implemented in the structure and the methods for data acquisition. The processing method is mainly electrical signals and algorithmic damage detection methods based on the collected data.

Data processing is shaped by the translation of the physical response data into electrical signals to transmit. In this process, the analog data collected and stored in the sensors is converted into digital electrical signals that are legible for the computer algorithm in the analysis stage. An integrated circuit (IC) is a vital component of this process, which has the capability to sense, translate, and communicate the data with other systems. These ICs typically consist of a sensor, an ADC, a microprocessor, and a connection to a data transmission medium. Micro electro-mechanical systems (MEMS) devices are recent advancements that fulfill this duty while existing on “a micrometer level” dimensionally. This technology orchestrates organized communication within the sensing network, the processing and transmission of data, and algorithmically determined energy efficiency within the sensors. A MEMS divides the processing algorithm into subsections to optimize the memory chip’s limited space. At the same time, other programmed ICs, specifically “smart sensors”, can dynamically allocate and free the data stored in the sensor to achieve this optimization. These processing units are continuously being researched and upgraded to optimize space, time, and energy consumption [7].

In the data analysis stage, a computer algorithm takes the sensor processing units’ output and translates the data into relevant results. This algorithm is responsible for deciphering the electrical signals into their corresponding mechanical responses and updating the virtual model of the structure. This way, the computer is in charge of filtering through the data, identifying irregularities, and locating possible damage to an area that may cause these changes in the response. There are many implementations of the damage recognition algorithm for sensing systems, varying from comparative data analysis to AI systems. In a comparative analysis, the input data from the sensing network is compared to pre-calculated and pre-tested structural response data of a healthy system. If the contrasted data shows major differences between the model and the measured response, the damage is reported within the structure. This, however, also requires extensive data on what a damaged structural response looks like to detect real damage within the system, as opposed to a sporadic response that may not represent physical damage. This can lead to complications and incorrect results by inputting data of various forms that may be irregular but do not reflect any damage to the structure.

Recent advancements to curb this problem on a long-term scale involve implementing artificial intelligence (AI) in the data analysis process. These AI systems are designed to be independent of model-data-based analysis and provide accurate damage detection and predictions of future deviations [45]. These systems use intense algorithms to determine the probability, location, and extent of damage within a structure. They are able to adapt to long-term changes within a structure’s mechanical responses.

## 5. Applications

### 5.1. Infrastructure, Construction, and Performance

There is a wide variety of applications for sensing systems in current and developing infrastructures around the world. The monitoring of public structures can help ensure their safety, reliability, and accessibility for common use, as well as prolong their service life. Infrastructures such as roadways, bridges, and large buildings may all benefit from the implementation of sensing systems.

In the U.S., the wood in transportation (WIT) program launched a campaign to construct over 75 wooden bridges between 1989 and 2004. The program is currently hosting an ongoing project, Development of Smart Timber Bridge, that implements SHM [46]. This project focuses on developing and advancing sensors for monitoring wooden structures under loads and environmental changes. These sensors would be able to successfully detect damage and generate alerts whenever maintenance is required to extend the service life of the bridges. In Norway, moisture, temperature, humidity, and strain sensors were installed on five wooden bridges to monitor the structural properties under varying conditions [47]. The sensing system was in operation for approximately nine years, with only two necessary system recalibrations, and showed no significant changes in measurement accuracy, verifying the long-term reliability of the monitoring system. In Sweden, a network of smart sensors was implemented on three timber bridges (two road traffic and one pedestrian) to measure the moisture content [48,49]. The SHM system applied to the pedestrian bridge was assembled to incorporate wireless communication between the sensors and the database. The findings of this system showed data corruption and data loss during communication when using wireless sensors working at the same frequency that is in the same or adjacent structures. The limited capabilities of wireless data communication highlight the need for advancements within smart sensing systems before their reliability can be verified.

In pavement infrastructures, such as roadways, the road conditions and their constituents can be monitored using a sensing network [50]. Some sensors are even capable of measuring traffic using weight-in-motion systems that can detect the speed, weight, and size of vehicles, with a measurement accuracy of over 90% [51]. These sensing systems must be constructed with temperature and humidity sensors to properly calibrate the data and monitor the road conditions, as the mechanical properties of pavement vary greatly concerning different temperatures and moisture contents. By measuring these properties, it is possible to detect and predict hazard conditions such as icy or slippery roads, as well as accurate traffic measurements.

### 5.2. Heritage Buildings

Heritage buildings are long-term structures that require preservation for their value (historic, cultural, etc.). These structures are often post-tensioned or reinforced with high-strength materials to preserve their structural health over time. The implementation of sensing systems in these buildings can assist their preservation and monitor the reliability of the post-tensioning of the structure. In Switzerland, the House of Natural Resources, located on the campus of ETH Zurich, installed an extensive sensing network and was subject to vibration-based tests during and after completion [52]. The sensing network measured a wide variety of structural and environmental properties, including temperature, humidity, deformations, stress, strain, and modal vibrations. The application of these sensors required complex integration and costly equipment, resulting in only a short period of measurements.

In New Zealand, the Trimble Navigation Office was reconstructed, after being destroyed by natural disasters, as a post-tensioned building [53]. The monitoring system installed aimed to ensure the long-term behavior of the post-tensioning system and verify its reliability in a high seismic risk area. The monitoring system remained operating for three years and collected data on the building’s response to normal operating conditions and intermediate tectonic activity. Overall, the application of sensing systems in this building was able to validate that the post-tensioning system operated according to the design assumptions, providing aid to the mechanical response of a long-term structure.

### 5.3. Construction Materials

The application of sensing systems on construction materials depends on what parameters are of interest for each distinct material. Damage that can be unique to each respective material require different methods of detection, while some universal damage can use similar approaches. The method applied to a material must be planned and calibrated in order to accurately measure the structural health.

Palma & Steiger (2020) [1] discuss the various techniques applied for respective damage in wooden structures, which primarily focus on measuring the changes in a structural response. Biological decay, fungi, and insect infestation are some of the damage commonly attributed to wooden structures. The method of assessing this damage must be chosen after careful consideration, as they are mainly found in wood and not equally in other structural materials. Other damage, such as internal cracking, strain, and deformation, which are also common to other materials, can be measured using a sensing system. There is a wide selection of sensors and methods to choose from, and, in many cases, different methods can be used to measure the same property.

In pavement applications, deformation and internal damage can be monitored through model-related analysis of the mechanical response of a given structure. This application of a sensing system attempts to measure crack width and propagation through indirect measurements collected by vibrational-based sensors [51]. The sensing system is also capable of detecting other common damage, such as permanent deformities, potholes, and surface defects. Measuring the strain on the pavement allows for not only the detection of damage, but also physical causes of damage based on what the pavement structure is qualified to support. These sensors can be embedded into the pavement, given that they have adequate packaging to prevent damage to the sensor or its performance. They can also monitor internal health, environmental parameters, and external forces.

The application of sensing systems in common construction materials allows for monitoring the foundational components of larger structures. The data collected from these sensors can be used to schedule any necessary rehabilitation of these materials, as well as monitor their quality before their implementation to ensure the reliability of a structure.

## 6. Sensing System Limitations

### 6.1. Sensor Network Metrological Limitations

The deployment of sensor networks brings about various metrological challenges that require careful consideration to ensure the reliability and accuracy of the collected data. One key limitation arises from the inherent variability among sensors within the network. Differences in manufacturing, calibration, and aging processes can result in variations in sensitivity and response characteristics among individual sensors. These discrepancies, if unaccounted for, may lead to inconsistencies in measurements across the network, affecting the overall quality of the data. Environmental conditions also pose significant challenges to sensor networks. Factors like temperature changes, humidity levels, and electromagnetic interference can introduce uncertainties in sensor readings. For instance, temperature fluctuations can affect the material properties of sensors, leading to changes in their electrical characteristics. Humidity may impact the performance of certain types of sensors, particularly those susceptible to moisture. Electromagnetic interference from nearby electronic devices or power sources can introduce noise into the sensor signals, further complicating accurate measurements.

Calibration drift is another metrological concern. Over time, sensors may deviate from their initial calibration due to various factors such as wear, aging, or environmental exposure. Periodic recalibration becomes essential to maintain the accuracy of measurements, but the frequency and methods of recalibration must be carefully determined to ensure the sustained reliability of the sensor network. Communication delays and synchronization issues among sensors within the network can contribute to temporal inaccuracies in data collection. Efficient data transmission and synchronization protocols are crucial to maintaining the temporal integrity of measurements, especially in applications that require real-time monitoring.

Researchers and engineers continually work on developing advanced calibration techniques, robust sensor designs, and sophisticated data processing algorithms to address these metrological limitations. As sensor networks become increasingly integral to various fields, from smart cities to industrial automation, overcoming these challenges is essential to unlocking the full potential of these interconnected systems [54,55,56,57,58].

### 6.2. Energy Supply

For sensing systems to detect, translate, and transmit data, the sensors and related hardware must be connected to a power supply. Finding a viable, long-term power supply for these systems is one of the key limitations of their autonomous implementation. Many of the common power source applications, such as battery power or solar systems, are not suited for long-term use and require periodic replacement. Other methods, such as connecting the system directly to the power grid, may result in energy inefficient and impractical applications. The energy requirements and data accuracy of a sensing system depend on the frequency of measurements taken and the energy needed to operate specific hardware. Reducing these parameters may extend the service life of the sensors but, alternatively, compromise the reliability of the data [51].

Sensing systems can have a variety of implementations that contribute to their energy usage. Active monitoring systems are equipped with sensors and actuators, where power is delivered to the actuators to apply an excitation, such as vibration, to the system. The sensors then detect the structure’s response, and the parameters are measured [1]. The functionality of the actuators is crucial for the implementation of an active monitoring system, such that these require a reliable power source for consistent excitations. The power needed for the sensors and actuators varies depending on what type of parameter is measured and excitation is used (vibrational, optic, etc.). While this method supplies reliable data, it can ultimately be an inefficient application of energy resources compared to other implementations. Passive monitoring systems, on the other hand, are typically implemented to measure a single parameter under normal environmental conditions, such as temperature or humidity [59]. This implementation does not require deliberately applied excitations (actuators), as the sensors make use of the “ambient excitations” of a structure [1]. Semi-active monitoring systems take periodic measurements for a short amount of time, where the system is activated by some external trigger [60]. The semi-active systems are triggered when some external excitation of the system passes a certain threshold detected by an accelerometer [51]. The sensors of the localized area are then activated to collect their designated measurement. If any damage is detected, it can be localized by activating all sensor nodes and evaluating the collective data. These semi-active systems use less energy and are more practical than the active monitoring systems but come with issues of understanding “how, when, and for how long” the sensors need to be powered [51].

An alternative method to powering the sensing systems, apart from battery power, is known as energy harvesting. In this practice, piezoelectric transducers convert the ambient energy of an environment into electrical power to be used by the system [61]. Typically, this method is implemented in a semi-active monitoring system, where the external trigger delivers energy to the transducer, which will then power the sensors. While in theory this method may reduce or eliminate the need for external power sources, in practice, the transducers do not supply enough energy for reliable data measurements. Graziano et al. (2020) [51] communicate that the power converted by the piezoelectric hardware is “characterized by inconsistent voltage spikes” that cannot power the sensors enough to collect, process, and transmit the data for the necessary amount of time. This highlights the need to find a method for the accumulation and optimization of the harvested energy for the most efficient times to activate the sensors.

While they may reduce the energy usage of a sensing system, the most significant limitation of semi-active systems is the ability to quickly take measurements and transmit the data in a limited amount of time. The sensors must be able to turn on and detect the structure’s response in a timely manner in order to collect reliable data. Furthermore, in wireless sensing networks, the sensor must be able to transmit the data while still being powered, so that there is no data loss during the transfer [7]. Both of these processes must be performed as quickly as possible in order to use the least amount of energy necessary.

Overall, there are many different implementations of sensing systems that require various power levels. Finding a method for the highest energy efficiency without compromising the accuracy of the collected data is crucial to constructing a reliable monitoring system. Energy consumption is not, however, the only factor contributing to the sensors’ service life and functionality.

### 6.3. Packaging

The sensors must be protected against their exterior without the loss of functionality, especially when the sensing system is to be integrated into a structure exposed to harsh environments. Many sensors are designed to be either embedded within a structure during the construction process or inserted in already existing structures. The packaging of these sensors must be strong enough to withstand the construction and/or structural environment and prevent damage to the hardware. Especially when sensors are integrated during construction, the packaging must be able to withstand elevated temperatures and service loads while keeping the functionality of the sensor intact. Xu et al. [62] reported that the change of temperature variation affects the baseline of the conductance signature of the sensor as well as the frequency and amplitude of the resonance peaks in the conductance signature. Traditional strain sensors are often encapsulated in metal packaging, but in recent implementations with wireless communication, this can interrupt the data signal transmission between the sensor and the server. The proper use of epoxy resin has been proved to protect the hardware from high pressure and heat while allowing efficient energy harvesting and data transmission [63].

The packaging of a sensor must be able to fit into small corners, gaps, and beams, all while maintaining its sensitivity and avoiding damage to the structure. Graziano et al. (2020) [51] evaluate the various shapes of sensor packages that relate to their operations and placement within a structure, where certain shapes (bone-shape, cylindrical, spherical) have different impacts on the functionality and protection of the devices. The use of the sensors in a system dictates the material, shape, and placement of the packaging that encapsulates them, as well as the structural environment they are implemented.

### 6.4. Network Layout

A major challenge when designing sensing systems is creating a network layout that associates the limited number of sensors with a possible number of positions for an optimum measurement [64]. The network must be efficient with the number of sensors implemented but must also not neglect any structural areas that may be subject to damage. The coverage, connection, and quality of the system are crucial for determining structural damage at an early stage [51]. The layout of a network is characterized by the elements, their roles, and their communication channels within a structure. Sensing networks connected by transmission cables are typically high in cost due to the expensive equipment and regular maintenance required. Recent advances in telecommunication have led to a widespread transition to wireless communication between sensors and central databases, which allows for flexibility and the self-organization of a sensing system [7].

In a wireless sensing network, the sensors communicate with a central node that relays the signals to a database to process the collected data. Graziano et al. (2020) [51] describe various connection layouts that can exist within a sensing network:

- Centralized: All sensor nodes in a network communicate directly to a single central node that relays the information to the database.
- Mesh type: Specific sensor nodes can communicate with one another, as well as the central node to transmit data.
- Middle way: Also called a “two-tier network”, the sensor nodes are divided into localized clusters, each of which has individual central nodes. These central nodes are connected to the central nodes of the other clusters over “far-range high data rate communication”.

The challenge associated with the network layout is finding an optimal configuration for a specific task or structure, which is not always universal. The system must be configured in such a way that maximizes efficiency for the unique structures they are implemented within. Additionally, with the possibility of expanding structures, the related sensing network and algorithms must also be able to be expanded proportionately [7]. While a larger number of sensor nodes equates to better data acquisition of a structure, this, in turn, raises issues with the energy efficiency and cost of the sensing network.

With the main goal of maximum data coverage with minimum hardware implementation, many studies have been carried out to utilize the data processing algorithm to compensate for areas in a structure that may be underrecognized by the sensing network. The “group effect” is an analysis strategy that interpolates the measured data of localized sensors to compensate for any unobserved areas [65]. This way, the known data can fill in the gaps for any areas that are not directly observed by the system, and the number of hardware elements can be reduced. This method, however, still has its own limitation, where the data accuracy is inversely related to the spacing between the local sensors.

The physical layout of the sensing system, paired with the relevant data processing algorithm, is designed and optimized for the structure to be measured. The specific aspects of and measurements to be taken on a distinct structure need a network layout capable of meeting the needs of the parameters to be monitored while aiming for maximum possible efficiency and data accuracy.

### 6.5. Performance Validation

The effectiveness of implementing a sensing system in a structure must be tested and verified in order to facilitate future research and applications. The performance of a sensing system can be validated through laboratory and in-field experimentation, typically tested in that order.

Laboratory testing utilizes simulations and small-scale experiments to verify that the sensing network should theoretically function properly. Simulations utilize 3D models and abstract loads to the structure in order to analyze and plan the best network layout [66]. This testing method is usually the base step in creating a physical sensing network, as sensor locations can be tested and optimized to work with the corresponding damage detection algorithm [51]. Simulations also offer insight into how loads can affect the performance of the piezoelectric transducers that deliver power to the semi-active systems. The simulation data, however, comes from calculated hypotheticals and is not reliable enough to validate how the system would respond in practice. Small-scale laboratory testing is necessary to verify the behaviors of the key system components, such as “self-powering characteristics, survivability, wireless communication capacity, and … damage detection” [51]. These experiments validate the energy conversion of the transducers and the detection accuracy of the sensors before the construction and implementation of a full-scale network.

In-field experimentations are the implementation of a full-scale sensing system in a typically controlled environment to ensure the functionality and reliability of the sensing network’s data collection and transmission, as well as the algorithmic analysis of the collected data. This is known as the best validation method to assess the merits/demerits of the designed system [67]. The loads applied to the structure are typically known, in order to verify that the system response is accurate with the expected functional outcome. These tests usually span over just a few days and incorporate only known variables for functional verification of the system. These experiments, however, do not span long enough for the verification of long-term implementations of these monitoring systems.

### 6.6. High Variability of Physical and Mechanical Properties

The physical and mechanical properties of a structural material have high variability between both other structural elements and other members of the same element. Variability in these properties, also known as “defects”, can occur from semi-random variations in the anatomy of the element, and the degree of variability depends on the size and location of those defects related to the applied forces [1]. For example, the physical and mechanical properties of wood vary greatly from those of steel or concrete, but they also have significant differences from other pieces of wood due to their natural structure. These variabilities can cause inconsistencies in the structural integrity and the calibrations of the applied sensing network of a structure. To compensate for these variabilities, materials for structural applications can be categorized based on their strength. This categorization leaves a high probability of variations between the physical and mechanical properties of structural members but allows for some consistency. These variations can limit the sensing system as the data collected may not be uniform for the individual structural members, and the network should be calibrated accordingly.

### 6.7. Moisture and Temperature Dependency

The response of a sensing system may vary depending on the temperature and moisture content of the surrounding environment [68]. The system must be calibrated to measure and compensate for these variables in order to ensure reliable data that may change significantly with these factors. The moisture and temperature of an environment can have a strong influence on the data collected, causing variations in the structural response and sometimes even the data transmission, such as in fiber optic sensors.

In timber applications, the moisture content of the wood has a strong effect on the member’s structural integrity, as the internal moisture favors equilibrium with the humidity and temperature of the external environment [69]. Within a wooden structure, moisture-induced stresses can form within the structural member as a result of smaller members adjusting to the surrounding environment faster than larger members, creating moisture gradients in the structure. The strength and stiffness of a wooden member are inversely related to the moisture content, and moisture stresses can eventually lead to mechanical failures within the structure.

In pavement applications, however, the moisture content is less of a concern than the temperature of the environment, which predominantly affects the response of the structure when subject to loads [70]. The stress and strain response of the pavement is greatly dependent on the temperature, and it is crucial to integrate a temperature sensor in the system to account for and calibrate these differences. The moisture content of pavement can also be monitored to predict hazardous conditions, such as slippery roads or significant moisture levels that could indicate damage to the structure.

### 6.8. Duration-of-Load Effects

The duration-of-load effects refer to the change in the strength of a structural member due to the long-term subjection of the member to loads. The effects depend on the type and magnitude of the loads, but overall, the longer the load is applied, the more the strength of a member is reduced. Palma & Steiger (2020) [1] detail the strength reduction of wood to be between 40–80% over a ten-year period, depending on the composition, moisture content, and frame of the member. When subject to a load, a structural member undergoes both an instantaneous and permanent deformation. Depending on the conditions of the internal environment, the load, and stress applied to a member, the permanent deformation, also known as creep, can deepen or relax and stabilize. These deformations in a structure can change the data in the sensing system and leave room for error in the damage detection process. With deepening deformations, the system’s response to certain loads can change and ultimately cause complications within the sensing system.

## 7. Influential Factors

### 7.1. Machine Learning

Machine learning (ML) can make sensing system applications very versatile and widespread. They facilitate the ability of complex simulations as well as the performance of certain tasks by analyzing numerous similar trial examples [71]. A machine learning algorithm uses model data and data collected over time to compare to current data and diagnose any significant results. These systems are able to operate without human intervention, where they can adapt to operate to a wide variety of responses, given proper model data, and can reconstruct algorithms for greater accuracy.

A crucial component of machine learning, an artificial neural network (ANN), is the combination of hardware, processing units, and software that create a complex data network that allows AI systems to grow and learn [72]. The hardware units and processing layer include the components necessary to build a sensing system network with structured communication between the DAQ and DAS. The software layer of an ANN organizes the data into structured data graphs that highly resemble neuronic systems in a human brain. The network can identify data trends and patterns and train the AI algorithm to remember and notice them for damage detection. Deep learning (DL) is another crucial component of machine learning, that works closely with the ANN [73]. The DL algorithms, compared to ML without its implementation, allows for an algorithm to analyze data without the need for structured or labeled data. The DL system consists of an algorithm that can sort through and recognize patterns or trends in the data and make inferences of what typical data should resemble. This way, there is no need for example data to be generated and fed into the computer to analyze and make assumptions about the data.

### 7.2. AI-Assisted Systems

AI-assisted systems use a complex machine learning algorithm to monitor the data reported from the structural network. Damage detection in these systems can operate with and without model data, whereas in the model-free method, development of a complex structural model is not required. The computer uses pattern and trend recognition to make assumptions about the past, present, and future conditions of a structure’s health and responses. The model-based method, on the other hand, requires extensive example data on the response of a healthy structure and of a wide variety of damage that may arise, as to properly detect which defect is present. This system may, however, provide more accurate reports on the type and extent of damage in a structure in the early stages of data collection. At the same time, the model-free method will still be able to detect damage but may not be capable of determining what damage is present. Over time, the machine learning algorithm in a model-free approach will allow the computer to remember data patterns and trends and possibly label them on its own, providing similar results to a model-based method. A convolutional neural network (CNN) is a variation of an ANN that utilizes DL and imaging for pattern recognition in (primarily vibrational-based) structural monitoring [74]. The implementation of AI systems, along with smart sensors, breeds a highly intelligent and complex sensing system.

While a sensing system can be highly sophisticated, it can also be prone to errors within the sensing systems’ data detection, collection, and transmission that would affect the results of data analysis. The use of DL algorithms, as well as image processing algorithms, can facilitate abnormality recognition of a structure and its damage [7]. With this approach, the monitoring system may be able to more accurately identify the source of data anomalies, although it is highly complex. The AI-assisted system can, however, use the collected data and analysis as a method to gauge the health and functionality of a structure. Hybrid Intelligence systems, which consist of the cooperation between computational intelligence (CI), artificial intelligence (AI), and computer assisted learning (CAL), can be employed for further enhanced data analysis [7]. These systems can determine how probable the input data is for a structure and then analyze the approved data to filter through anomalies that are most likely to be errors within the sensing system. This improves the reliability of the sensing system to report the analysis of correct data and remove outliers that are, given the output of the plausibility check, not accurate representations of the structure.

### 7.3. Cloud Computing

Cloud computing networks utilize remote, off-site servers that organize and store data over the internet instead of local servers. The application of cloud computing servers allows for more complex algorithms for data analysis, as on-site servers would require larger and more hardware to complete the same tasks. The use of cloud computing also assists in data recovery that may be needed in the event of various damage, as on-site servers could be damaged, and data could be lost. Furthermore, the data stored in a cloud server can be accessible from different locations. The cloud servers also do not depend on on-site memory cards to store the data, and the database can be constructed and managed from a remote location. The cloud algorithm can also be continuously adjusted to cater to the amount of data or analysis speed of data parameters. This allows for greater accessibility and sizeability of a sensing system and its respective collected data [75].

### 7.4. Guidelines and Standards

Current guidelines of sensing system applications on structures focus on the monitoring procedures of bridges and offshore structures to avoid major intermissions for rehabilitation projects. Palma & Steiger (2020) [1] outline the various guidelines constructed for the structural monitoring of civil projects in different countries. Norwegian Technology Standards published the Condition Monitoring of Load-Bearing Structures in 1997, where they provide the general principles on how the monitoring of “offshore load-bearing structures should be planned, implemented, and documented”. The monitoring techniques described in this document are mainly visually based and manual measurements. In Germany, guidelines for the application, execution, and evaluation of measurements of longstanding structures are described in a document that remained published between 2000 and 2009 but was eventually withdrawn as the techniques related to mostly the technology of the publication time. ISIS Canada published Guidelines for Structural Health Monitoring—Design Manual No.2, which describes the field testing techniques (static and dynamic) for periodic (not continuous) monitoring of bridges. This publication describes the methodology for “selecting and protecting sensors”, “guidelines regarding data acquisition, and application examples”.

Other guideline reports build on these same previously mentioned topics but may include other standards that relate to the “design, calibration, and implementation of field measurement systems”, as described in The Smart Campus as a Testing Ground for Smart Cities, published by the American Society for Engineering Education [75]. The European Union funded a project to construct the Guideline for Structural Health Monitoring, which describes the assessment and modeling of loads acting on a structure, the methods and application of structural health monitoring and analysis, and damage identification and assessment techniques. The many guidelines for structural health monitoring that can be found today describe the general practices and techniques for various condition measurements and analyses to ensure a reliable and effective monitoring system.

## 8. Conclusions

The application of sensing systems in construction is a widely adaptable method for continuous overview and analysis of a structures functional value and need for any repairs or rehabilitation. There is an extensive selection of measurement methods for various mechanical or environmental properties of a structure. The many sensors and network connection modes between them can construct highly sophisticated monitoring systems and intelligent analysis methods.

Such systems utilize a virtual 3D model of the structure to be monitored, and sensors can be embedded within the structure and structural members for continuous data collection on the mechanical and physical properties. The different sensors that can be implemented in the sensing network have distinct functions and individual limitations. The individual sensing components are chosen by considering the effectiveness, cost, and longevity of the sensors when applied to a structure. The sensors must be protected and energy efficient while providing reliable data collection and transmission between the DAQ and DAS. Because of the variability of mechanical and physical properties based on other properties, such as environmental conditions, additional sensors may be needed in the sensing system to perform correct calibrations of the acquired data. These sensors must be protected from their external environment and suitable for long-term application in a structure.

The data analysis of a structure utilizes complex computer algorithms that extract relevant data and update the 3D model of a structure. Recent advancements have incorporated AI within the analysis phase, which is able to perform a more in-depth investigation of the provided data, with knowledge of previous data and predictions of future data. With AI-assisted systems, there may not be a need for a model or example data to compare to know what a healthy structural response or certain damaged responses may resemble. The use of these complex monitoring systems on a structure allows for the continuous supervision of the health of a structure and when restorations or rehabilitation may need to be scheduled.

The influential factors in sensing systems collectively contribute to their enhanced effectiveness and versatility. Machine learning (ML), particularly through the utilization of an artificial neural network (ANN) and deep learning (DL), stands out as a pivotal element, enabling complex simulations and damage detection without the need for structured data. AI-assisted systems offer a flexible approach to monitoring structural health, with both model-free and model-based methods. Cloud computing further enhances these systems by providing advanced off-site data analysis, ensuring recovery, and enabling accessibility from diverse locations. The guidelines and standards established by global organizations underscore the importance of monitoring procedures and offer comprehensive methodologies for structural health monitoring and analysis. Together, these factors signify a holistic and sophisticated approach to sensing systems, promising reliability, effectiveness, and adaptability in the realm of civil projects.
